# Relationship between anthropometric variables and nutrient intake in apparently healthy male elderly individuals: A study from Pakistan

**DOI:** 10.1186/1475-2891-10-111

**Published:** 2011-10-12

**Authors:** Iftikhar Alam, Anis Larbi, Graham Pawelec, Parvez I Paracha

**Affiliations:** 1 Tübingen Aging and Tumour Immunology group, Sektion für Transplantationsimmunologie und Immunohämatologie, University of Tübingen, Zentrum für MedizinischeForschung, Waldhörnlestraße 22, 72072 Tübingen, Germany; 2 Abdul Wali Khan University Mardan, Department of Agriculture, Khyber Pakhtunkhwa (Previously: NWFP), Pakistan; 3 Singapore Immunology Network (SIgN), 8A Biomedical Grove, IMMUNOS Bd.03, Biopolis, A*STAR, 138648, Singapore; 4 Department of Human Nutrition, Faculty of Nutrition Sciences, NWFP Agricultural University, Peshawar, Khyber Pakhtunkhwa (Previously: NWFP), 25000, Pakistan

## Abstract

**Background:**

The elderly population is increasing worldwide, which warrants their nutritional status assessment more important. The present study was undertaken to establish the nutritional status of the least-studied elderly population in Pakistan.

**Methods:**

This was a cross-sectional study with a sample of 526 generally healthy free-living elderly men (mean age: 68.9 yr; range: 50-98 yr) from Peshawar, Pakistan. Anthropometric measurements (weight, height, WC) were measured and BMI and WHR were calculated from these measurements following WHO standard procedures. Dietary intake was assessed by 24-hr dietary recall. Nutrients were calculated from the information on food intake. Nutrients in terms of % of RNI were calculated using WHO data on recommended intakes.

**Results:**

Based on BMI, the numbers of obese, overweight and underweight elderly were 13.1, 3.1 and 10.8%, respectively. Age was negatively and significantly correlated with BMI (*p *= 0.0028). Energy (*p *= 0.0564) and protein intake (*p *= 0.0776) tended to decrease with age. There was a significant increase in % BF with age (*p *= <0.0001). The normal weight elderly had significantly (*p *< 0.05) higher intake of all nutrients studied, except energy which was significantly (*p *< 0.05) higher in obese and overweight elderly. Overall, however, the majority of subjects had lower than adequate nutrient intake (67.3 - 100% of recommendation).

**Conclusions:**

Malnutrition is common in apparently healthy elderly Pakistani men. Very few elderly have adequate nutrient intake. Obese and overweight had higher % BF as compared to normal weight elderly. Older age is associated with changes not only in anthropometrics and body composition but also in intake of key nutrients like energy and protein.

## Background

There has been a rapid increase in the number of elderly people in Pakistan [1] hence maintaining health and well-being of this age group is becoming even more important. Beside so many other health risks associated with old age, this population is potentially the most vulnerable group for malnutrition [2]. Poor dentition, neuropsychological problems and immobility in older age directly affect their nutritional status [3].

The prevalence of overweight and obesity is increasing [4], particularly in the elderly [5], where it is associated with increased mortality and a number of metabolic and cardiac disorders [6]. Overweight and obesity also contributes to functional decline and disability in the elderly [7]. At the same time, quite significant numbers of old individuals are reported to suffer from underweight and are at higher risk for acute illness and death [8]. They also have significantly higher risk of dying within the first year of hospitalization than those with adequate nutrition [9]. Weight loss has been shown to be associated with a higher risk of disability [10]. Decreased body Mass Index (BMI) is an indicator of chronic energy deficiency and malnutrition, and is associated with compromised immune function, increased susceptibility to infectious illnesses, and reduced survival in the elderly [6].

Similar to other developing countries, Pakistan can be expected to experience the impact of an increasingly ageing population over the next few decades [1], with a steady rise in the average life expectancy from 59.1 years in 1991 to 65 years in 2002. This quite sudden demographic shift can be very challenging in terms of health and nutritional care. Essential information about individuals' food intake and habits, activity, cultural influences, and the economic and social situation provide a database for nutritional assessment. Developed countries have established dedicated health care systems in order to meet the special needs of the elderly. However, such programs are lacking in developing countries like Pakistan. To the best of our knowledge, so far no separate study has been undertaken to document the nutritional status of the elderly in Pakistan and this type of important information thus remains fragmentary or absent. Those nutritional surveys that have been conducted in the past, however, do show very marginal nutritional status and high nutrient deficiencies in the general population (not specifically the aged) [1]. In this context of higher prevalence of malnutrition in general population in Pakistan, it can be assumed that the elderly might have an even more impaired nutritional status. The present study, therefore, aimed to investigate the nutritional status and nutrient intake of Pakistani elderly. The results are expected to help in designing policies and making plans regarding health care provision for the elderly in Pakistan. Nutritional status is particularly worrisome in the context of the ageing population, which is becoming a serious demographic problem. Hence, elucidating the nutritional status of the elderly is of prime importance for formulating preventive strategies to lower morbidity rates, improve quality of life and reduce health care costs.

## Methods

### Study site and sample selection

The current study is a cross-sectional survey using focused interviews, conducted during 2008-09 in Peshawar, Pakistan. Participants of the study were elderly men from Peshawar in the province of *Khyber Pakhtunkhwa *(previously, the North West Frontier Province: NWFP) of Pakistan. In order to increase representation of the elderly, subjects were selected randomly from eight different sites in Peshawar. Women were not included mainly due to cultural constraints of the area. Taking into account the limited resources and time available, the convenience sampling method was adopted; recruiting a final total of 526 elderly men defined as ≥50 years of age. For our present work, we defined elderly as individuals ≥50 years of age partially based on the arguments of Glascock and Feinman (1980) [11], which provide a basis for definition of old age in developing countries. It is recommended to use change in social role (*i.e*. change in work patterns, adult status of children and menopause) as a criterion for definition of old age. We adopted this criterion as we observed that in Pakistan (and particularly in our study area) this social change in the life span starts at the age of around 50 years. For recruitment of the elderly subjects, city registration data were obtained from the local office of NADRA (National Database and Registration Authorities) in Peshawar. Addresses of the elderly subjects, who fulfilled the age and health criteria for the study, were obtained from the lists provided by NADRA.

### Data Collection

Data were collected by the first author assisted by trained graduate students of the Department of Human Nutrition, Agricultural University, Peshawar.

### Age and Anthropometric Data

Age was assessed using official documents (the National Identity Card, NIC). Weight and height were measured and BMI was calculated as weight/height^2 ^(kg/m^2^). Waist circumference (WC) and waist-to-hip ratio (WHR) are simple anthropometric indices for assessing the amount and distribution of body fat that can help in risk assessment for many health problems [12]. WC and HC (Hip Circumference) were measured according to the standard procedures reported in details elsewhere [13]. Briefly, WC was measured at the part of the trunk located midway between the lower costal margin (bottom of lower rib) and the iliac crest (top of pelvic bone) while the subject was standing with feet apart and weight equally distributed on each leg. The measurer (the first author) stood beside the individual and fitted a non-flexible tape snugly, without compressing any underlying soft tissues. The circumference was measured to the nearest 0.5 cm, at the end of a normal expiration. HC was measured with the same tape, placed around the point with the maximum circumference over the buttocks. The subject stood with feet fairly close together and weight equally distributed on each leg. The subject was asked to breathe normally and the reading of the measurement was taken at the end of normal expiration. The measuring tape was held firmly, ensuring its horizontal position. Due care was taken that the tape should be loose enough to allow the observer to place one finger between the tape and the subject's body.

Subjects were categorized into four groups as obese, overweight, normal weight and underweight based on their BMI values [2,4]. For assessment of central obesity, we used cut-off values of WC and WHR. Subjects with WC of <94, 94-101.9 and ≥ 102.0 cm were classified as normal weight, overweight and obese, respectively [2,4]. WHR (waist to hip ratio) was calculated as: WC/HC and subjects with WHR values of <0.90, 0.90-0.99 and ≥1.0 were classified as normal weight, overweight and obese, respectively. WC and WHR are not used to define underweight [2,4].

Percent body fat (%BF) of each subject was measured by Futrex-5000 according to the procedures recommended by the manufacturer (Futrex^®^, Hagerstown MD, USA). The device emits near-infrared light into the body at very precise frequencies (938 nm and 948 nm) at which body fat absorbs the light and lean body mass reflects it. From the amount of light absorbed and emitted the device calculates % BF. The measurements were taken at the midpoint of each participant's dominant bicep.

### Dietary Data

The dietary data were collected using 24-hr dietary recalls (24-hr DR) through face-to-face interviews conducted primarily in *Pashto*, the local language. These 24-hr DRs were repeated three times over the three alternative days of a week. No data, however, for Sunday (a weekly holiday in the study area) was collected. Because we observed in our pilot trial for validation of the 24-hr DR questionnaire that most of the subjects were away from homes for social reasons on Sunday and it was difficult for them to recall exactly what they had eaten when they were away. Nevertheless, this exclusion did not bias the results as our other analyses (data not shown) suggest that differences in nutrient intake over the weekend and weekdays were not significant in our study area, although some studies in other countries, for example the USA, have reported differences in nutrient intake over the weekdays and weekends [14]. During the 24-hr DR interviews, the intake reported by the subject was verified by someone in the household to avoid over- or under estimation of dietary intake because elderly might easily forget what they had eaten during the previous 24 hrs.

Household measures such as cups, bowls, and spoons were used to help estimate quantities of foods consumed. Quantities were recorded according to the amount of a particular bowl, for instance, 1/2 of the small brown bowl. When interviewees gave answers like, "I used a little or a lot of milk in tea", they were asked to show this with the cup they used, and the cup volume was later measured to estimate the amount. Nutrient intakes were computed using an in-house nutrient calculator (Microsoft Office Excel 2003, USA). This calculator is based on the data from food composition tables for Pakistan [15]. Mean and standard deviation (SD) of energy, protein, selected minerals (Ca, Fe, Zn) and vitamins (A and C) were determined from dietary intake data. The vitamins and minerals selected are those known to be important, particularly for the older population [16]. Reference Nutrient Intakes (RNI) of the World Health Organization/Food and Agriculture Organization (WHO/FAO) [17] were used because Pakistan has no nutrient recommendations of its own. The percentage of elderly with adequate nutrient intake was ascertained. Nutritional adequacy for each nutrient was calculated by comparing the actual intake with the recommended values for a nutrient. For most of the nutrients, recommendations are usually set about 30% above the average requirement in order to cover the need of almost all healthy people of the respective sex and age group [18]. For this reason, it has been customary to use a cut-off value of two-thirds (66.7%) of the recommended intake to estimate the proportion of a population with adequate intakes [18]. Therefore, adequate consumption was considered to be 66.7-100% of the RNI for a particular nutrient.

### Statistical Analysis

All anthropometric measurements were made in duplicate and the means of paired values were used in the analyses. The data were statistically analyzed using JMP (Version 7.0. SAS, USA). As the current study involved four BMI categories, the means of nutrient intake in these four BMI categories (*i.e*. obese, overweight, normal weight and underweight) were taken for one-way analysis of variance (ANOVA), and post-hoc comparisons with Dennett's test taking the normal weight group as reference. BMI-adjusted partial correlation coefficients were calculated to establish associations between anthropometric measurements and nutrient intake. The resulting *p*-values demonstrate significance or lack thereof. The cut-off points used were: p ≥ 0.05 is a non-significant difference and p < 0.05, a significant difference.

The current study was approved by the Board of Studies, Department of Human Nutrition, Agricultural University Peshawar. Written informed consents were obtained from all the participants before the start of study.

## Results and Discussion

The present study included only apparently healthy individuals with no recent past or present smoking or any other drug addiction history. Table 1 shows general and socio-demographic characteristics of the study subjects. Table 1 also shows % number of elderly in four BMI categories and mean (SD) % BF of elderly in these BMI categories. As evident, more than half (51%) of study subjects were illiterate and relatively a high number (82%) were living with their families. Based on BMI, there were 13.1, 3.1, and 10.8% obese, overweight and underweight elderly, respectively. The mean (SD) % BF ranged from 15.5 (6.41) to 38.4(7.21), respectively in the underweight and obese elderly.

**Table 1:** General and anthropometric characteristics of the study subjects

Mean age (yrs)	68.9 (8.80); Range: 50 - 98 yr
Education (% number of subjects )	
*Primary*	24
*High*	8
*Others (non-conventional)^1^*	17
*Illiterate*	*51*
% number of economically active^2^	41
% number living with families	82
% number whose wives had died	48
% number in four BMI groups ^3^	
*≥ 30 *	13.1%
*24.9 - 29.9 *	3.1%
*18 - 24.9 *	73.0%
*<18 *	10.8%

Mean (SD) % BF in four BMI groups	
*Obese*	38.4 (7.21)
*Overweight*	32.2 (5.18)
*Normal Weight*	25.6 (5.52)
*Underweight*	15.1 ( 6.41)

^1^Non-conventional refers to the particular education system imparted in local *Madrassas *(the religious education system in Pakistan).^2^Economically active refers here to an engagement in a job or service for earning purpose.^3^BMI categories as per WHO (2003)

Table 2 shows % number of overweight and obese elderly defined by BMI, WC and WHR. Most of the overweight and/or obese elderly defined by any of these three criteria were in the age group of 60.1 - 70 yr. Based on BMI, WC and WHR, 8.6, 4.9, and 29.2% elderly were either overweight or obese in this age category; the highest as compared to other age categories. The other age category with the second highest percent prevalence of obesity and/or overweight was 70.1-80 yr. The prevalence of WHR-defined obesity was the highest (23.2%) in the age group 60.1 - 70 yr. Furthermore, in all age groups WHR gave the highest prevalence of obesity followed by BMI- and WC-defined obesity. These results show that either BMI or WC alone may underestimate the prevalence of obesity in elderly and, therefore, WHR may be a stronger and more sensitive indicator for estimation of obesity and/or overweight in epidemiological studies. These results further show that in elderly central or abdominal obesity (assessed by WC or WHR) may be more prevalent than general obesity (assessed by BMI).

**Table 2:** Percent of overweight (OW) and obesity (OB) by body mass index (BMI), waist circumference (WC) and waist-hip ratio (WHR) cut-offs

Age (yrs)	N	BMI		WC		WHR	
		OW	OB	OW	OB	OW	OB
50-60	59	0.7	0	1.3	0.2	4.7	1.1
60.1-70	260	6.2	2.4	3.8	1.1	23.2	6
70.1-80	154	3.1	0.9	1.5	0.4	9.3	1.5
80.1-90	65	0.4	0	0.7	0	4.7	0.7
>90	7	0.2	0	0.4	0	1.6	0.2

Overall	526	10.6	3.3	7.7	1.7	43.5	9.5

BMI = Body Mass Index; WC = Waist Circumference; WHR = Waist to hip ratio; OW = Overweight; OB = Obese

Table 3 presents the mean daily intake of selected nutrients by elderly stratified by BMI groups. There were large differences in nutrient intake comparing all the three groups (*i.e*. obese, overweight and underweight) to the normal weight group. Obese and overweight elderly seemed to be consuming significantly (*p *< 0.0001) more energy than people of normal weight but significantly less protein, calcium, iron, vitamins A and C. Further, the results show that underweight elderly had significantly lower mean intake of all nutrients studied as compared to the normal weight elderly (*p *value ranged from 0.0001 - 0.0006).

**Table 3:** Mean (SD) of nutrient intake in four BMI categories

Nutrients	Obese (OB)	Over-weight (OW)	Normal weight (NW)	Under-weight (UW)	p-value^1^		
					*OB-NW*	*OW-NW*	*UW-NW*
Energy (Kcal)	2266 (312.2)	2058 (219.5)	1651 (311)	817 (312)	<0.0001	<0.0001	<0.0001
Protein (g)	41.8 (6.68)	42.3 (6.79)	43.4 (6.41)	27.0 (7.06)	0.002	0.0421	<0.0001
Fiber (g)	6.8 (1.62)	7.6 (2.06)	9.4 (1.60)	3.5 (1.14)	0.0481	0.0041	<0.0001
Calcium (mg)	342.4 (79.1)	392.2 (91.6)	451.4 (111.1)	270 (83.1)	<0.0001	0.0052	<0.0001
Iron (mg)	11.2 (2.48)	12.7 (3.5)	13.1 (2.81)	7.2 (2.90)	0.0139	0.0139	<0.0001
Zinc (mg)	7.3 (1.31)	7.2 (1.7)	7.5 (1.58)	4.4 (1.18)	0.1421	0.0411	<0.0001
Vit A (RE)	283.6 (97.2)	298.3 (113.1)	314.9 (194)	219 (106.5)	0.0439	0.0501	0.0006
Vit C (mg)	32.3 (17.3)	25.9 (13.7)	44.4 (12.3)	14.2 (8.16)	0.0431	0.0411	<0.0001

^1^. p-values were calculated using Dennett's test in JMP. The normal weight castigatory was considered as reference. Alpha value for significance was 0.05

**Figure 1:**
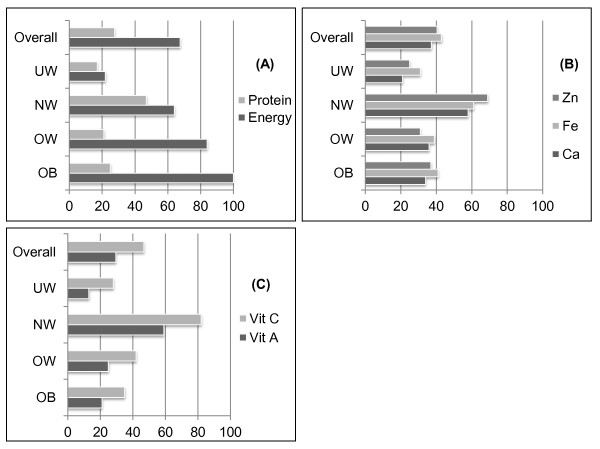
**Percent (%) Number of elderly in four BMI categories with adequate intake of nutrients**. The adequate intake is defined as intake 67.3 - 100% of the recommended intake

The % number of elderly with adequate nutrient intake in each BMI category is depicted in Figure 1. Overall, very few elderly had adequate energy and protein intake. In obese and overweight categories, 100 and 84% of the elderly had adequate energy intake, while very few people in those two categories had adequate protein intake. Similarly, in the normal weight and underweight BMI categories, adequate energy and protein intake were reported for 64 and 22, and 47 and 17%, respectively. Similarly, for minerals and vitamins, even lesser than 45% of the elderly in obese, overweight and underweight categories had an adequate intake of Ca, Fe, Zn, vitamin A and vitamin C. As expected, the percentage of normal weight elderly with adequate intake for these nutrients was higher than either of the other BMI categories.

One encouraging fact was that the participation rate in this study was fairly high (73.6%). Because subjects in poor health are often not able and also not willing to participate, selectivity in favor of subjects in better health can hardly be avoided in studies involving the elderly. The same holds true for poorly-educated persons [19].

The nutritional assessment of free-living elderly in Pakistan in the present study has demonstrated the need to promote a healthy lifestyle in this population. BMI, WC, WHR, and % BF measurements showed that most of the elderly people had abnormal nutritional status with very high energy intake in the obese category and inadequately lower energy intake in the rest of the BMI categories. The need for the elderly to improve their nutritional status and balance their dietary intake has been a long-standing topic of discussion among nutritionists. Many studies have associated higher energy intake with obesity and overweight and lower energy intake with body decomposition, which may result in a decreased DNA repair capability, lower plasma glucose levels, diminished insulin sensitivity and overall unhealthy lifespan [6,19].

In current study, all the anthropometric variables were included on the basis of their association with food habits, health and well-being in the elderly [20]. Weight reflects the recent and present balance between energy utilization [21]. Height/stature reflects genetic potential and nutritional status during growth and is also related to fat-free or lean body mass, which is a good index of protein stores [22]. BMI calculated from weight and height [23] is related to percentage of body fat and to fat-free mass, while WC and HC are useful indices of adipose tissue and central obesity [24].

The present study highlights an alarmingly high prevalence of overweight, obese and underweight even in relatively healthy and wealthy Pakistani elderly men, measured either by BMI, WC or WHR. In particular, very high numbers (43.6%) of elderly were found to be either overweight or obese assessed by WHR (Table 2), which is especially important in view of the fact that Asian adults have higher cardiovascular risk factors already at lower BMI and WC than Western populations [16]. These arguments may support the fact that alone BMI is not enough to determine the risk of developing obesity-related conditions. Excess abdominal fat, regardless of overall body fat, will predispose to obesity-related disease. This highlights the importance of measuring WHR. It is possible that two persons with very similar BMI may vary substantially in the proportion of abdominal fat. Accordingly, a person with a BMI in the "normal" weight range may exceed the safe range of abdominal fat. In aged individuals with a decline in lean muscle mass, their BMI may not change or may even decrease, but fat levels could increase with the accompanying redistribution of body fat. WHR and WC are useful and reliable measures of abdominal obesity but both of them have their individual strengths and weaknesses and both are usually measured in a clinical evaluation.

In addition, BMI has also been criticized for its poor discrimination between fat and muscle mass. Thus, those individuals who are overweight not because of an increased amount of body fat, may have a high BMI value, but should not be considered obese. There are data indicating that even though BMI is a reliable measure of fatness in children and young individuals [25], an adolescent's percentage of fat can change by as much as -3 to +7% without any difference in BMI. For an individual adult, the same BMI can correspond to changes in fat of ±5% [26]. Additionally, BMI seems to have a reduced applicability to the elderly [27]. For this very reason, WC and WHR are used for better discrimination of obesity, particularly the central or abdominal obesity [24,26,28]. However, all these anthropometric measurements have certain limitations [29] and therefore, cannot be used in isolation to predict results.

Data on nutritional status of elderly is also very fragmentary in Pakistan. Other studies documenting the prevalence of obesity and overweight in the elderly seem essentially absent. There has been no nationwide study to document the prevalence of obesity in the other population groups either. Some small-scale local studies, however, reported variable rates of overweight and obesity in Pakistan [30]. Higher prevalence of obesity and/or overweight in Pakistani population with increasing age has also been reported previously [30,31]. The results of these studies are in close agreement with ours, finding the highest mean measurements of BMI, WC and WHR in the elderly age group of 60.1-70 yr. The difference in prevalence as reported by the current and the previous studies might be mainly due to difference of age of the sample, sample size and sample characteristics.

In current study, we found fewer elderly had adequate nutrient intakes (Figure 1). Energy intake seemed to be adequate (66.7-100% of the recommended intake) in 100, 84 and 64%, respectively of obese, overweight and normal weight elderly, but only in 22% of the underweight elderly. The overall number of elderly individuals with adequate energy intake was 67.5%, which means more than 33% were energy-deficient and had inadequate (<66.7% of the recommended intake) energy intake.

The prevalence of energy deficiency in Pakistan is not unexpected [32], particularly in the elderly [33]. If BMI < 18.5 kg/m^2 ^is used as an indicator of chronic energy deficiency in the elderly [34], prevalence of chronic energy deficiency as high as 13.1% is reported in the current study. Low BMI values in relation to low energy intake in Asian elderly populations have also been reported in the IUNS Study [35]. Even in developed countries, data show a high prevalence of energy deficiency in the elderly [36]. Lower energy intake causes body decomposition [18]. On the other hand, due to problems with mastication and poor dentition [33,37], elderly prefer caloric-dense foods with proportionally limited amounts of other necessary nutrients, which might be a contributing factor to age-related obesity and deficient intake of other important nutrients.

In current study, protein intake in all four BMI categories seemed to be inadequate (Table 2). Only very few elderly had adequate (66.7-100% of the recommendation) protein intake in the four BMI categories (Figure 1A): 25, 21, 47, and 17% of the obese, overweight, normal weight and underweight elderly, respectively, with an overall of 27.5%, had adequate intake. This implies that a large proportion (72.5%) of the elderly had inadequate (<66.7% of the recommendation) protein intake. Requirements for protein in the elderly are still under debate [31]; but it is quite safe to say that there was a high risk of protein deficiency in our study group of the elderly.

The % number of elderly in the four BMI categories with adequate Ca, Fe, Zn (Figure 1B) and vitamin A and vitamin C (Figure 1C) intake ranged from 21 - 58% for Ca; 31 - 61% for Fe; 25 - 69% for Zn; 13 - 59% for vitamin A and 28 - 82% for vitamin C. However, the overall numbers of elderly with adequate intake of these nutrients were only 37, 43, 41, 30, and 47%, respectively. To the best of our knowledge, there have been no separate data on the intake of these nutrients by Pakistani elderly. However it has been reported that mean intake of Ca, Fe and Zn by adults in the general Pakistani population is much lower than the recommendations [38]. Mean calcium, iron, and zinc intake in the present study seemed well within the intake range of most countries [39]. However, the % number of subjects with adequate intake of these nutrients was very low.

It is also noteworthy that most nutrients consumed by the elderly in the present study were derived from plant sources (data not shown). This intake pattern is similar to that in many other developing countries [40], which may be one of the reasons for deficiencies in certain nutrients in this age group. For example, phytates present in whole-grain breads, cereals, legumes and other plant foods bind zinc and inhibit its absorption [41]. Factors found mainly in plant foods including phosphorus, flavonoids, oxalates and soy protein can also inhibit iron absorption and decrease its bioavailability [42].

**Figure 2:**
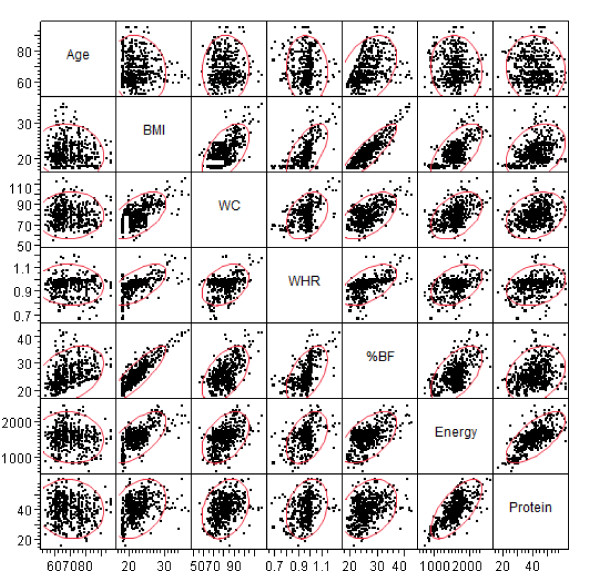
**Correlation Matrix**. The correlation analysis was performed for age, anthropometric measurements (BMI, WC, WHR,), %BF, energy and protein. The alpha level of significance is 0.05.

The correlation analyses (Figure 2) show that with increasing age there was a significant decrease in BMI (*p *= 0.0028; *r *= -0.1304). Energy (*p *= 0.0564; *r *= -0.1236) and protein intake (*p *= 0.0776; *r *= -0.0771) tended to decrease with age but not significantly, while a non-significant increase in WC (*p *= 0.3124; *r *= 0.0422) and significant increase in % BF (*p *= <0.0001; *r *= 0.3655) with age were noted. Unlike WC, WHR decreased with age. However, this decrease was not significant statistically (*p *= 0.1220; *r *= -0.0675). Studies show a decrease in BMI with age, particularly after 60 yr [43,44], an increase in fat mass [45] and a decrease in energy intake [36]. However, these changes are very variable [43-45]. Nevertheless, all these associations of selected anthropometric measurements and nutrients with age are important from the aging and nutrition point of view as an understanding of the underlying factors affecting body composition may facilitate correction by simple nutritional interventions. An increase in body fat with aging may be partly attributed to a loss in muscle mass, even in independently-living healthy subjects [27]. Furthermore, skeletal muscle mass loss in men is masked by weight stability, resulting from a corresponding increase in total body fat mass. Progression of sarcopenia, particularly in men, may therefore be clinically silent and comparable to the loss of bone mineral density in osteoporosis [27].

In conclusion, there is a high prevalence of underweight, overweight and obesity in elderly Pakistani men. We report a limitation of prediction made either by BMI, WC or WHR alone as a measure of overweight and obesity, based on our results and the published literature. The nutritional data demonstrated that majority of subjects had a suboptimal nutrient intake. We propose that the current BMI-based categories be reviewed for the Pakistani population, particularly for the elderly. Furthermore, we suggest that BMI, WC and WHR should be used in combination to define nutritional status. In addition, we suggest that attention should also be paid to the problem of underweight in old age.

## Competing interests

The authors declare that they have no competing interests.

## Authors' contributions

IA and GP designed research; IA, and PIP conducted research and collected the data; IA and AL analyzed the data; IA wrote the manuscript; Critical revision of the manuscript for important intellectual content was the responsibility of IA, AL and GP. IA had full access to all the data in the study and takes full responsibility for the integrity of the data and the accuracy of the analysis. All authors read and approved the final manuscript.
